# Supplementary material for: A model of ganglion axon pathways accounts for percepts elicited by retinal implants
Source: Sci Rep. 2019 Jun 24;9:9199. doi: 10.1038/s41598-019-45416-4 (PMC6591412; doi:10.1038/s41598-019-45416-4)
Supplement: Supplementary file 1 — Supplemental Information [file 41598_2019_45416_MOESM1_ESM.pdf]

A model of ganglion axon pathways accounts for percepts elicited by retinal implants

Michael Beyeler[1,2,3], Devyani Nanduri[4], James D. Weiland[4,5], Ariel Rokem[2,3], Geoffrey M. Boynton[1], and Ione Fine[1,*]

[1]Department of Psychology, University of Washington, Seattle, WA 98195, USA

[2]Institute for Neuroengineering, University of Washington, Seattle, WA 98195, USA

[3]eScience Institute, University of Washington, Seattle, WA 98195, USA

[4]Department of Biomedical Engineering, University of Southern California, Los Angeles, CA 90033, USA

[5]Department of Biomedical Engineering, University of Michigan, Ann Arbor, MI 48109, USA

*Correspondence: mbeyeler@uw.edu

Supplemental Information

S1. Threshold measurement

Perceptual thresholds were used to determine the suprathreshold level of stimulation (2x threshold) used for the drawing task described in the main text.

S1.1 Argus I subject

Perceptual thresholds were measured using custom-developed software on single electrodes using an adaptive yes/no procedure. Stimuli for measuring threshold were charge-balanced, 0.45 ms / phase, cathodic-first, biphasic 20 Hz pulse trains, 500 ms in duration. There was no interphase interval (i.e., the total cathode-anode pulse width was 0.90 ms). On each trial, subjects were asked to judge whether or not they saw a phosphene on that trial. Half of the trials were stimulus-absent catch trials interleaved randomly with the stimulus-present trials. Current amplitude was varied using a three-up-one-down staircase procedure to find the threshold current amplitude needed for the subjects to see the stimulus on 50% of stimulus-present trials, corrected for the false alarm rate. During each staircase, only amplitude varied while all other parameters (frequency, pulse width, pulse train duration, and the number of pulses) was held constant. Each threshold was based on fitting a Weibull function to a minimum of 125 trials and error bars are estimated using Monte-Carlo simulation (Wichmann and Hill, 2001).

S1.2 Argus II subjects

Perceptual thresholds were measured using custom-developed software using a yes/no procedure that was a hybrid between an adaptive procedure and a method of constant stimuli. Stimuli were charge-balanced, 0.45 ms / phase, cathodic-first, biphasic 20 Hz pulse trains, 250 ms in duration. As in the Argus I stimulation, there was no interphase interval (i.e., the total cathode-anode pulse width was 0.90 ms). However, in the Argus II procedure, rather than testing each electrode individually, as many as six different electrodes were tested in a single experimental run. The entire experimental run was divided into five separate blocks of 12 trials per electrode in each block for a total of 72 stimulation trials per block. In addition to stimulation trials, 32 catch trials in total were interspersed randomly over the five presentation blocks. Thus, the maximum number of trials over 5 blocks was 72×5+32 = 392 trials. Stimulus amplitudes (for stimulus present trials) for the first block were predetermined (method of constant stimuli). After the first block, a maximum likelihood algorithm fit of a Weibull function to the current data determined the range of the next block of stimulation amplitude values for each electrode. After each block a confidence interval was acquired for each electrode using a Monte-Carlo simulation based on responses to the previous trials. If the confidence interval for an electrode fell below to a pre-set level, trials for that electrode were no longer presented, but trials on the other electrodes continued through a maximum of five blocks.

Results were deemed unreliable if the false alarm rate, determined by the percentage that the subject saw a stimulus during catch trials, was greater than 20%. Data from runs with higher false alarm rates than 20% were removed from analysis and the runs were repeated.

S2. Tactile target control task

Our experiments relied on the ability of our subjects to draw percepts accurately and consistently across trials. However, our blind subjects lacked tactile-visual feedback for many years. As a result, we were concerned that they might be unable to reliably reproduce visual phosphenes.

We therefore carried out a control experiment with tactile targets to establish baseline drawing errors for each subject. For the Argus I subject (Subject 1), the test stimuli consisted of a set of 11 tactile shapes made of felt with a cardboard background (**Figure S1A**). For Argus II subjects, methods were refined to use six tactile shapes made of felt with a cardboard background (**Figure S1B**). Subjects were asked to feel the felt shapes (**Figure S1C**), and then draw them on a board or a touch screen.

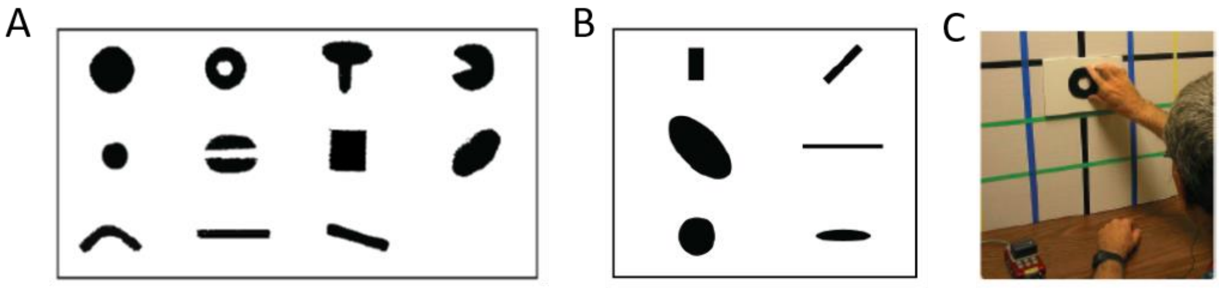

Figure S1: Tactile target control task. (**A**) Argus I tactile shapes. (**B**) Argus II tactile shapes. (**C**) Subject feels shape before tracing on a screen.

Shapes were classified in terms of their area, their orientation, and the major and minor axis lengths calculated from raw image moments. Visual inspection of the drawings suggested that subjects may differ in their drawing ability between compact and elongated shapes. We therefore subdivided the data into two subgroups (‘elongated’: major axis length larger than twice the minor axis length, equivalent to elongation $> \sqrt{0.5}$ in the main text), and plotted drawing errors separately.

We calculated drawing errors as the differences across repeated drawing trials for a given tactile target (**Figure S2**). Subject 1 showed less area variability across trials for compact (17±2% error) than for elongated shapes (34±2% error). Orientation was less variable for elongated (8°±2° error) than for compact shapes (22°±4° error). Subject 2 showed similar area variability for compact (20±5% error) and elongated shapes (17±9% error). Orientation was less variable for elongated (6°±3° error) than for compact shapes (23°±20° error). Subject 3 showed comparable

area variability for compact (20±8% error) and elongated shapes (23±3% error). Orientation was less variable for elongated (8°±2° error) than for compact shapes (22°±4° error). Subject 4 showed less area variability for compact (17±2% error) than for elongated shapes (34±2% error). Orientation variability was comparable for compact (9°±9° error) and elongated shapes (8°±6° error).

We then calculated drawing bias as the ratio or difference between the tactile target and the mean shape of drawings of that tactile target (**Figure S3**). Subject 1 drew both compact and elongated shapes larger than the actual size of the tactile targets (compact: 1.5±0.15 times larger; elongated: 1.8±0.3 times larger), and tended to draw elongated shapes biased by 9°±1.85° counter-clockwise. Subject 2 drew both compact and elongated shapes smaller than the tactile targets (compact: 0.46±0.11 times smaller; elongated: 0.56±0.09 times smaller), and tended to draw elongated shapes biased by 16°±5.41° counter-clockwise. Subject 3 drew both compact and elongated shapes larger than the tactile targets (compact: 1.5±0.31 times larger; elongated: 2.4±0.19 times larger), and tended to draw elongated shapes biased by 4°±1.7° clockwise. Subject 4 drew both compact and elongated shapes larger than the tactile targets (compact: 2.5±0.36 times larger; elongated: 2.3±0.68 times larger), and tended to draw elongated shapes biased by 14°±4.3° counter-clockwise.

Overall, trial-by-trial variability in the phosphene drawing experiments was similar to that of the tactile experiments— consistent with the data in the main paper suggesting that electrically stimulated percepts might be relatively consistent across trials.

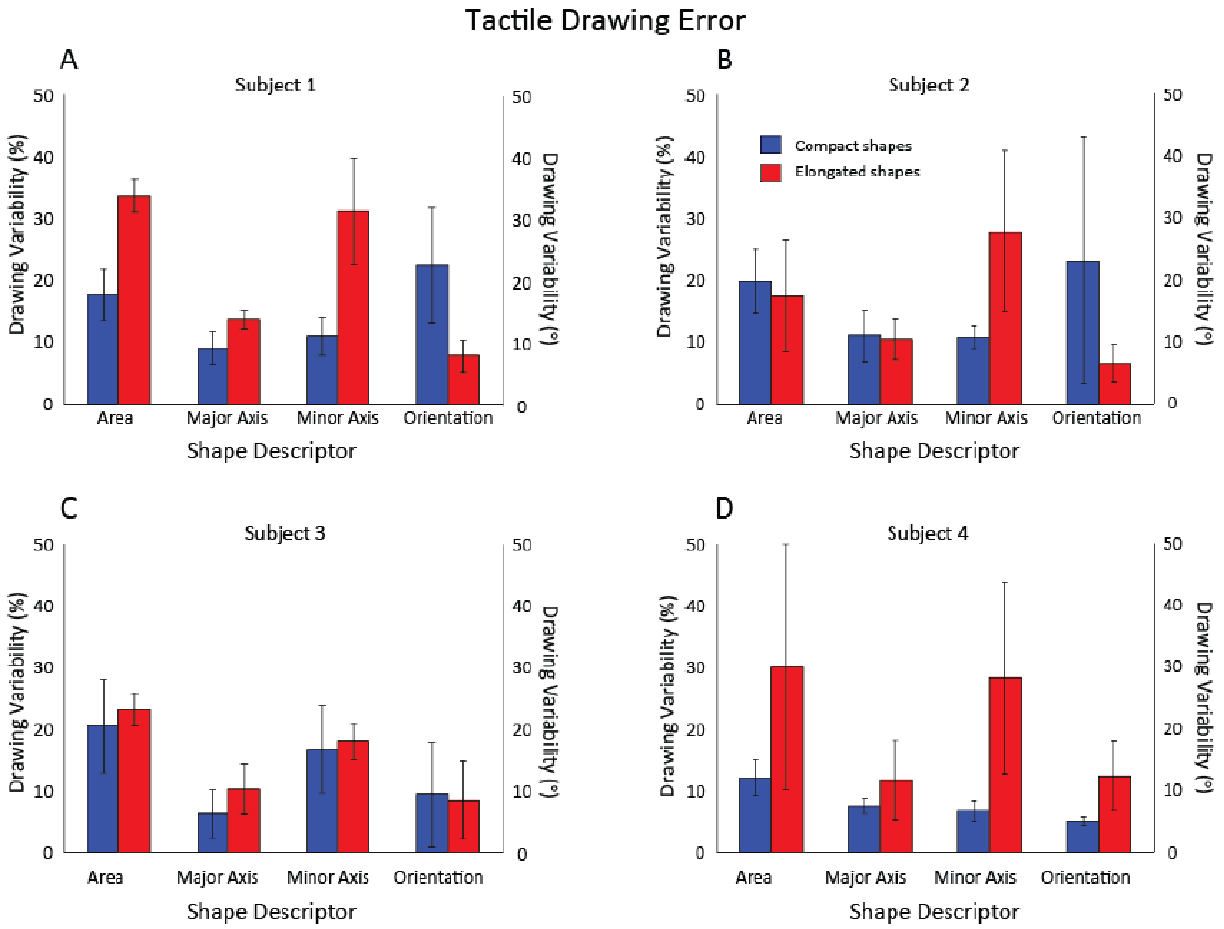

Figure S2: Tactile drawing errors for all subjects.

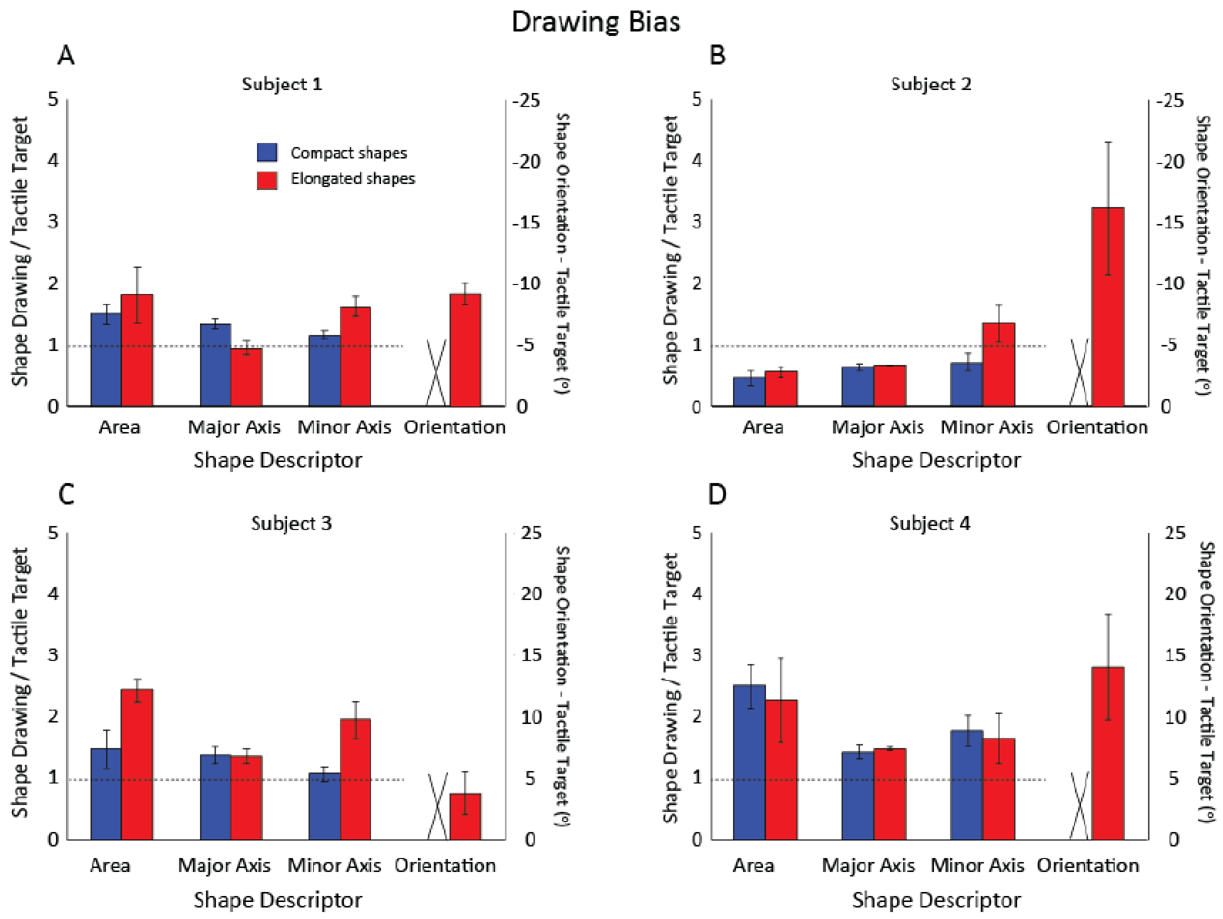

Figure S3: Tactile drawing biases for all subjects.

S3. Area vs. elongation

Figure S4 shows the correlation between phosphene area and elongation. For all but Subject 3, elongation was negatively correlated with area; meaning that smaller phosphenes tended to be more elongated than larger ones. This result held when data from all subjects were grouped together ($r = -0.411, p < 0.001$).

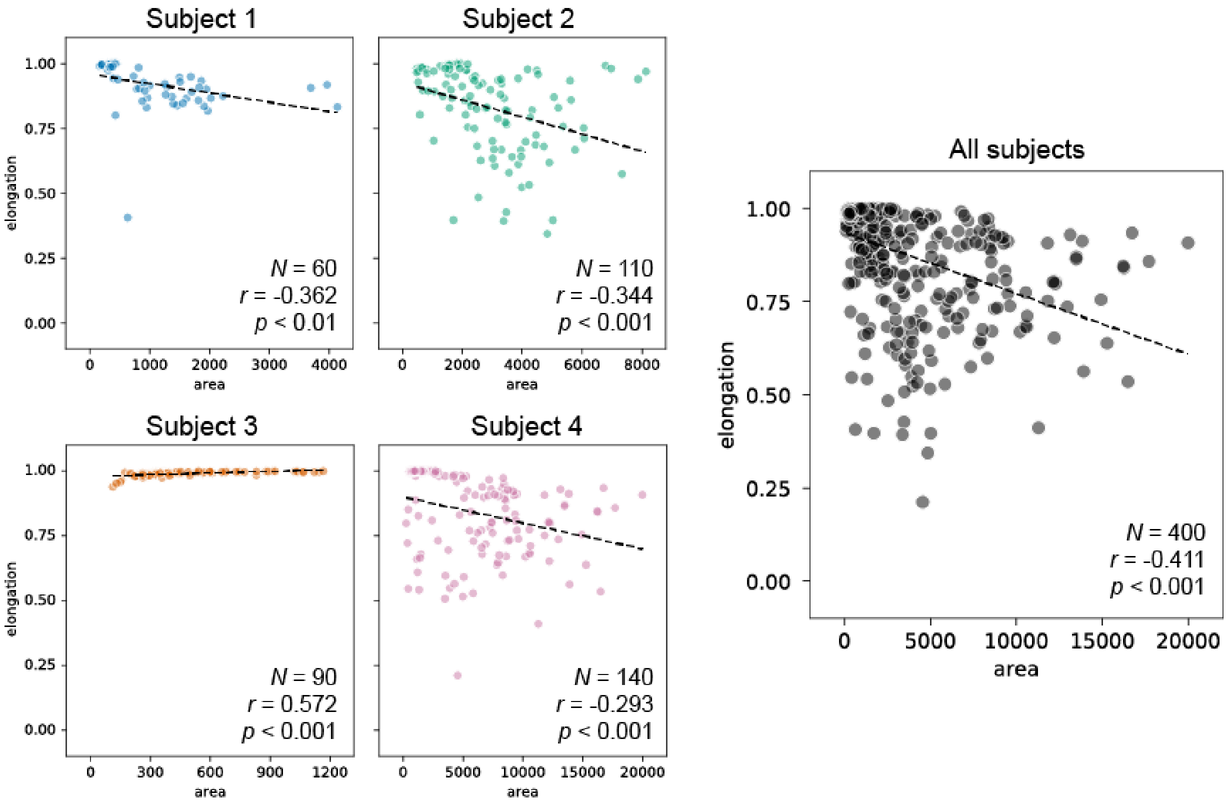

Figure S4: Correlation between phosphene elongation and area. Each data point represents the extracted elongation and area of a single phosphene drawing. Scatter plots are shown for each subject (S1-S4; small panels) as well as grouped across subjects ('all'; large panel). N: number of data points, r: correlation coefficient, p: p-value.

S4. Supplementary references

Wichmann, F.A., and Hill, N.J. (2001). The psychometric function: II. Bootstrap-based confidence intervals and sampling. Perception & psychophysics 63, 1314-1329.
